# Evidence That Regulation of Pri-miRNA/miRNA Expression Is Not a General Rule of miPEPs Function in Humans

**DOI:** 10.3390/ijms22073432

**Published:** 2021-03-26

**Authors:** Anne Prel, Christine Dozier, Jean-Philippe Combier, Serge Plaza, Arnaud Besson

**Affiliations:** 1 Laboratoire de Recherche en Sciences Végétales, UMR5546 CNRS, UPS Université de Toulouse, 31320 Auzeville-Tolosan, France; prel.anne@lrsv.ups-tlse.fr (A.P.); combier@lrsv.ups-tlse.fr (J.-P.C.); 2 Molecular, Cellular and Developmental Biology Department (MCD), Centre de Biologie Integrative (CBI), University of Toulouse, CNRS, UPS, 31062 Toulouse, France; arnaud.besson@univ-tlse3.fr

**Keywords:** miPEP, pri-miRNA, miRNA, short ORFs, micropeptides

## Abstract

Some miRNAs are located in RNA precursors (pri-miRNAs) annotated as long non-coding (lncRNAs) due to absence of long open reading frames (ORFs). However, recent studies have shown that some lnc pri-miRNAs encode peptides called miPEPs (miRNA-encoded peptides). Initially discovered in plants, three miPEPs have also been identified in humans. Herein, we found that a dozen human pri-miRNAs potentially encode miPEPs, as revealed by ribosome profiling and proteomic databases survey. So far, the only known function of plant miPEPs is to enhance the transcription of their own pri-miRNAs, thereby increasing the level and activity of their associated miRNAs and downregulating the expression of their target genes. To date, in humans, only miPEP133 was shown to promote a positive autoregulatory loop. We investigated whether other human miPEPs are also involved in regulating the expression of their miRNAs by studying miPEP155, encoded by the lnc MIR155HG, miPEP497, a sORF-encoded peptide within lnc MIR497HG, and miPEP200a, encoded by the pri-miRNA of miR-200a/miR-200b. We show that overexpression of these miPEPs is unable to impact the expression/activity of their own pri-miRNA/miRNAs in humans, indicating that the positive feedback regulation observed with plant miPEPs and human miPEP133 is not a general rule of human miPEP function.

## 1. Introduction

microRNAs (miRNAs) are small non-coding RNA of around 22 nt in length, identified in various species, which, by binding to target mRNAs, mediate their degradation and/or inhibit their translation. MiRNAs play key roles in various developmental and physiological processes, and their deregulation has been linked to pathological disorders such as cancer, diabetes, and obesity [1,2]. In this regard, they have emerged as biomarkers and potential therapeutics. MiRNAs are generated from the cleavage, in the nucleus, of long primary transcripts (pri-miRNAs) to generate ~60–80 nt precursors (pre-miRNAs) that are exported to the cytoplasm and further processed into mature miRNAs. Pri-miRNAs are transcribed by RNA polymerase II, capped, spliced, and polyadenalyted. Recent works performed using a novel experimental and computational approach have allowed a better annotation of pri-miRNA transcript structures [3]. While many miRNAs are hosted within well-characterized protein-coding genes, a subset of them are located in RNAs annotated as long non-coding (lncRNAs), given the lack of long protein-coding open reading frames (ORFs). However, recent advances in genomics, ribosome profiling, and proteomics have revealed that numerous lncRNAs harbor short ORFs (sORFs) encoding small peptides that have important biological activities [4,5]. Interestingly, in plants several putative lnc pri-miRNAs were shown to contain functional sORFs that code for regulatory micropeptides called miPEPs (miRNA-encoded peptides) [6,7,8,9]. These miPEPs enhance the transcription of their own pri-miRNAs, thereby increasing the level of their associated miRNAs that downregulate the expression of their target genes in plant cells [7,8]. Consequently, by impacting miRNA regulation networks, miPEPs were shown to affect the growth and development of various plant species [6,7,8,10]. Until now, very few miPEPs have been identified in animals. Studies performed to identify micropeptides encoded by sORFs-containing lncRNAs involved in the immune response in humans, showed that the lncRNA MIR22HG (pri-miRNA-22) codes for a peptide (C17ORF91) induced in response to viral infection [11]. However, the biological function of this peptide has not been investigated yet. Other studies have shown that human pri-miRNAs can encode miPEPs, and identified sORFs in the pri-miRNA of miR-200a and miR-200b [12]. Overexpression of these sORFs-encoded peptides (miPEP200a and miPEP200b) in prostate cancer cells inhibits their migration by downregulating vimentin expression [12]. Since miR-200a and miR-200b also regulate cell migration [13], it was suggested that the miPEPs200 might function by activating miR-200a and miR-200b, as in plants. However, the mechanism by which miPEPs200 regulate vimentin expression has not been elucidated. Recently, human lncRNA MIR155HG (pri-miRNA-155), an important regulator of hematopoiesis, inflammation, immunity, and tumorigenesis [14], was reported to encode a 17-amino acid micropeptide named miPEP155, which was shown to suppress autoimmune inflammation by regulating antigen transportation and presentation in antigen-presenting cells [15]. However, whether miPEP155 can regulate the expression of its pri-miRNA/miRNA was not determined. Another miPEP, miPEP133, was recently identified as a 133 amino acid peptide encoded by pri-miRNA 34a. MiPEP133 is expressed in various normal tissues and downregulated in cancer cell lines and tumors, and was shown to function as a tumor suppressor in cellulo and in vivo when overexpressed [16]. Interestingly, miPEP133 localizes to mitochondria and enhances p53 transcriptional activity by disrupting mitochondrial function. Since MIR34AHG, encoding pri-miR-34a/miR-34a, is a p53-target gene, this results in an increase of pri-miR-34a/miR-34a expression [16].

Despite evidence that pri-miRNAs code for miPEPs in human cells, it remains unaddressed whether the regulation of miRNA gene expression observed with plant miPEPs and with human miPEP133 is a general rule of human miPEPs. To address this question, we focused on three miPEPs: miPEP155, encoded by the lnc MIR155HG [15]; miPEP497, a sORF-encoded peptide within lnc MIR497HG; and miPEP200a encoded by the pri-miRNA of miR-200a and miR-200b [12]. We show that overexpression of these miPEPs does not impact the expression/activity of their own pri-miRNA/miRNAs in humans, suggesting that the positive feedback regulation observed with plants miPEPs and with human miPEP133 is not a general rule of human miPEPs function.

## 2. Results

### 2.1. The Pri-miR-155 and Pri-miR-497 Transcripts Are Translatable in Hela Cells

The plant pri-miRNAs were shown to contain translatable sORFs by fusing miPEP ORFs with the GUS (β-glucuronidase) reporter gene [7]. First, we investigated whether human pri-miRNAs are translatable. To avoid difficulties of interpretation with protein-coding genes, we focused on pri-miRNAs (MIRHG for miRNA host gene) identified as lncRNAs in UCSC Genome Browser. Using ribosome profiling as a first indication of translation marks, we analyzed the 48 promising candidates with GWIPS-viz (Genome Wide Information on Protein Synthesis visualized), an online genome browser for viewing ribosome profiling data [17] (http://gwips.ucc.ie (accessed on 4 March 2021)). We found that among the 48 candidates, 32 MIRHGs contained potentially translated sORFs, highlighted by ribosome profiling marks (Table S1), suggesting that translatable sORFs within pri-miRNAs are widespread in humans. Moreover, a study performed on pri-miRNAs of exonic miRNAs showed that some spliced pri-miRNA transcripts present a cytoplasmic localization [18], consistent with translation. Many lnc MIRHGs exhibit a complex gene structure and are expressed as multiple transcript variants due to alternative promoter usage and/or alternative splicing [3]. In the present study, we focused on MIRHGs with the least complex structure, such as MIR155HG and MIR497HG. 

**Figure 1:**
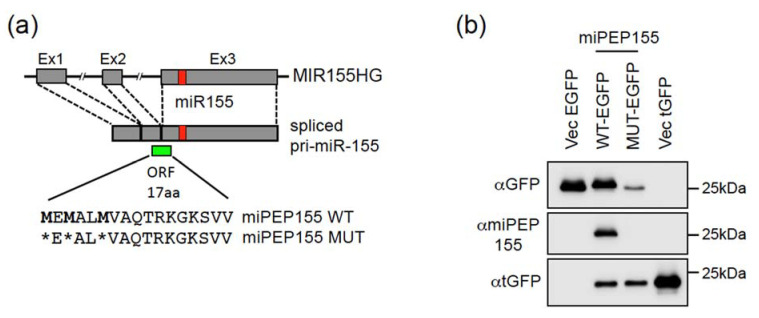

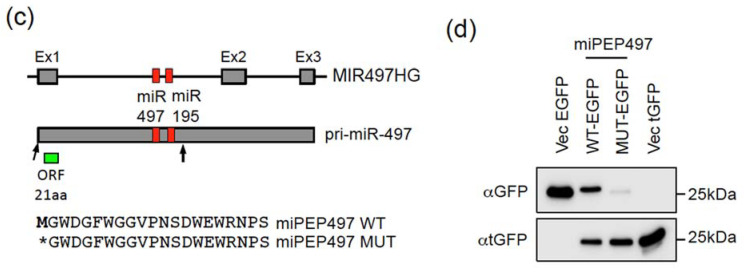
The pri-miR-155 and pri-miR-497 transcripts are translatable in Hela cells. (**a**,**c**) Schematic structures of human MIR155HG and pri-miR-155, (**a**) and MIR497HG and pri-miR497, (**c**) with the miRNAs (red boxes), sORFs (green boxes), and miPEP sequences indicated. *: mutation of ATG into stop codon. (**b**,**d**) Hela cells transfected with vectors expressing either the EGFP (Vec-EGFP) or turboGFP (Vec-tGFP) proteins or wild-type (WT) or mutated (MUT) miPEP155-EGFP (**b**) or miPEP497-EGFP (**d**) fusion proteins were analyzed by Western blot with the indicated antibodies. The vectors expressing miPEPs-EGFP proteins also express the turboGFP (tGFP) to monitor the transfection efficiency.

MIR155HG, also known as BIC for B-cell integration cluster gene, is a well-characterized gene encoding miR-155 and is expressed as unspliced or spliced pri-miRNA transcripts (Figure 1a), which are both used for miR-155 processing [18,19]. While unspliced transcripts are located almost exclusively in the nucleus, spliced transcripts are present both in the nucleus and in the cytoplasm [18,19]. The pri-miR-155 exhibits marks of ribosome profiling on exon2 and at the beginning of exon3 (Figure S1), which could correspond to a translated sORF of 54 nt (sORF54). This sORF is located 5′ of pri-miR-155 and could code for a peptide of 17 amino acids in length (Figure 1a). In agreement with this, MIR155HG was recently reported to encode a 17-amino acid micropeptide named miPEP155, detected in HEK293T, OCI-LY-1 (human B cell lymphoma), and human dendritic cells [15]. The miPEP155 is extremely well conserved in primates and partially conserved in mice (Figure S2). Since ribosome profiling marks of exon2/exon3 were also detected in Hela cells [20], we determined whether the sORF54 was translated in Hela cells. To this end, we constructed a vector expressing the full-length spliced pri-miR155 isoform with the wild type (WT) or ATGs-mutated (MUT) sORF54 (Figure 1a) placed in frame with the EGFP (Enhanced Green Fluorescent Protein) coding sequence lacking its start codon. Transfection of these miPEP155-EGFP constructs in Hela cells showed that the WT construct is translated into a fusion miPEP155-EGFP protein, detected both with an anti-GFP and a specific antibody produced against miPEP155 (Figure 1b). These results indicate that the spliced pri-miR-155 transcript is translatable and able to express miPEP155 in Hela cells.

According to Ensembl Genome Browser (http://www.ensembl.org, (accessed on 4 March 2021)), MIR497HG, hosting miR-497 and miR-195, is expressed as unspliced and spliced transcripts. Only the unspliced transcript, which we termed pri-miR-497, can be used for miR-497 and 195 processing (Figure 1c). The first exon exhibits marks of ribosome profiling (Figure S1), corresponding to a sORF of 66 nt (sORF66), which could code for a 21-amino-acid-long peptide, called miPEP497 (Figure 1c), which is also well conserved in primates and mice (Figure S2). To determine whether the start codon of sORF66 is active, we constructed a vector containing the pri-miR-497 sequences, spanning from the 5′ end to 53 nt downstream miR-195 (delimited by arrows in Figure 1c), with the WT or ATG-mutated sORF66 cloned in frame with EGFP lacking its start codon. Transfection of these miPEP497-EGFP constructs in Hela cells showed that the WT construct is translated into a fusion miPEP497-EGFP protein, detected with an anti-GFP antibody (Figure 1d), indicating that the nucleotide context of the sORF66 is in a favorable translational context. Thus, the pri-miR-497 transcript contains at least one translatable ORF located upstream of the miR-497 and is able to express a miPEP in Hela cells.

In summary, our results and the recent reports [11,15,16] reveal that among 32 potentially translatable MIRHGs according to ribosome profiling experiments, four out of four tested (MIR155HG, MIR497HG, MIR22HG, and pri-miR-34a) were experimentally validated. 

### 2.2. The miPEP155 and miPEP497 Do Not Regulate the Levels and Activities of Their Pri-miRNAs/miRNAs

**Figure 2:**
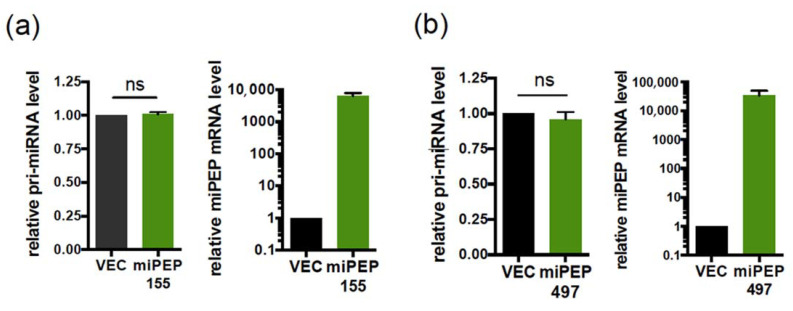
The miPEP155 and miPEP497 do not regulate the levels of their corresponding pri-miRNA. (**a**,**b**) Left: Expression of endogenous pri-miR-155 (**a**) or pri-miR-497 (**b**) upon overexpression of miPEP155 (**a**) or miPEP497 (**b**) into Hela cells. Pri-miRNA levels, quantified by quantitative RT-PCR, were normalized to GAPDH and set to 1 for the control vector (VEC) transfected cells. Data are means ± S.E.M. of three (**a**) or four (**b**) independent experiments. The right panels confirm the overexpression of miPEPs in transfected Hela cells. ns: not significant.

In plants, it was initially shown for two miPEPs that they enhance the transcription of their own pri-miRNAs [7], a concept further extended to other miRNA genes and in various plant species [8,10]. So far in humans, only miPEP133 has been shown to promote such a positive autoregulatory loop [16]. To determine whether this is a general mechanism, we investigated the potential regulatory role of miPEP155 and miPEP497 on the expression of their pri-miRNAs. Hela cells were transfected with either the control vector (VEC) or the miPEP expression constructs, and the levels of endogenous pri-miR-155 and pri-miR-497 were quantified 48 h post-transfection by quantitative RT-PCR. Results show that, while we detected the expression of miPEPs constructs at the RNA levels (Figure 2a,b, right panels), there was no change in endogenous pri-miR-155 (Figure 2a, left panel) or pri-miR-497 (Figure 2b, left panel) levels upon overexpression of miPEP155 or miPEP497, respectively. 

**Figure 3:**
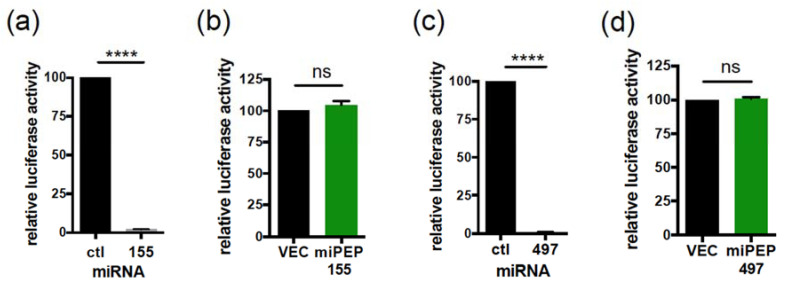
The miPEP155 and miPEP497 do not regulate the activity of their corresponding miRNA. (**a**,**c**) miR-155 (**a**) and miR-497 (**c**) activities upon overexpression of miR-155 (**a**) or miR-497 (**c**) into Hela cells. (**b**,**d**) Endogenous miR-155 (**b**) and miR-497 (**d**) activities upon overexpression of miPEP155 (**b**) or miPEP497 (**d**) into Hela cells. Cells were tested 48 h post-transfection for dual luciferase assays. (**a**,**c**) The relative luciferase activities of miRNA transfected cells were compared to that of the miR ctl transfected cells, set to 100. Data are means ± S.E.M. of three independent experiments. (**b**,**d**) The relative luciferase activities of the miPEP transfected cells were compared to that of vector transfected cells, set to 100. Data are means ± S.E.M. of four independent experiments. ****: *p* < 0.0001. ns: not significant.

We next questioned a possible regulatory role of miPEPs on the activity of their endogenous associated mature miRNAs. To do this, we constructed sensors of miR-155 and miR-497 activity obtained by cloning a miR-155 or miR-497 synthetic target into a dual luciferase reporter vector. Co-transfection of the miR-155 or miR-497 luciferase reporters together with a control miRNA or their corresponding miRNAs into Hela cells showed a significant reduction in luciferase activities with the miRNAs relative to control miRNA (Figure 3a,c), even at low concentration of miRNA (Figure S3a,b), validating the sensitivity of our miRNA sensors. Co-transfection of Hela cells with miR-155 sensor together with an anti-miRNA control or with an anti-miR-155 inhibitor able to rescue the overexpression of miR-155 (Figure S4a) revealed that endogenous miR-155 is active in Hela cells (Figure S4c). Similar experiments performed with the anti-miR-497 inhibitor revealed a weaker but significant detectable activity of endogenous miR-497 in Hela cells (Figure S4b,d), in agreement with a previous report [21]. However, co-transfection of miR-155 or miR-497 luciferase reporters together with a control vector or miPEP expression constructs showed that overexpression of miPEP155 or miPEP497 did not affect endogenous miR-155 (Figure 3b) or miR-497 (Figure 3d) activities, respectively. 

**Figure 4:**
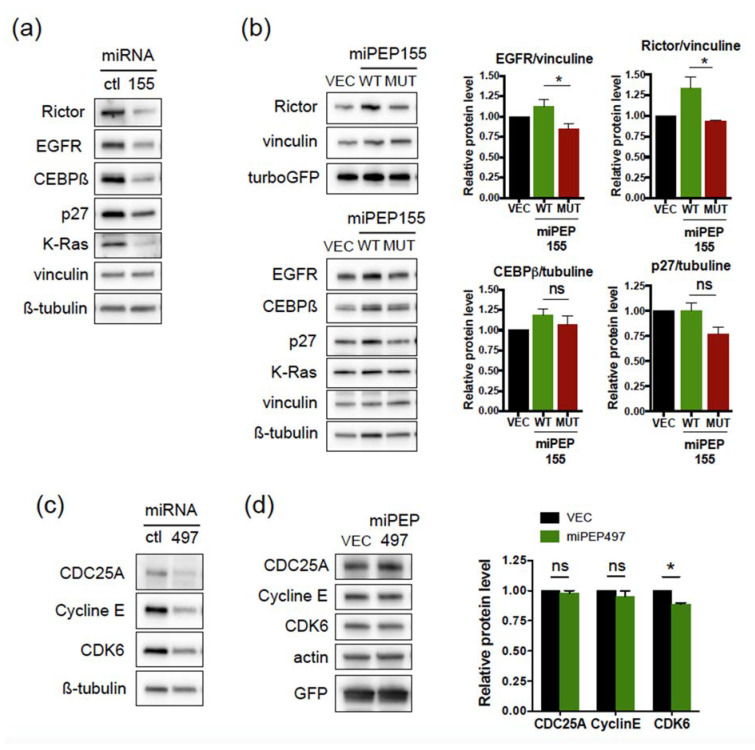
miPEP155 and miPEP497 do not regulate their corresponding miRNA target genes. (**a**,**b**) Hela cells transfected with control miRNA or miR-155 (9 nM) (**a**) or control vector or vectors expressing WT or mutated miPEP155 (**b**, left panel) were analyzed by Western blots 48 h post-transfection using antibodies directed against the indicated proteins. Vinculin and ß-tubulin were used as loading controls. The vectors contain the turboGFP gene expressed from the same mRNA, used as control of transfection efficiency. (**c**,**d**) Hela cells transfected with control miRNA or miR-497 (10 nM) (**c**) or control vector or vector expressing miPEP497 (**d**) were analyzed 48 h post-transfection by Western blots using antibodies directed against the indicated proteins. Actin was used as loading control. The vectors contain the EGFP gene, used as control of transfection efficiency. (**b**,**d** right panels) Quantification of the immunoblots. Data are means ± S.E.M. of four independent experiments. * *p* < 0.05. ns: not significant.

Analysis of the expression of miR-155 and miR-497 target genes supported these data. Overexpression of miR-155 in Hela cells led to a downregulation of Rictor, EGFR, CEBPß, K-Ras, and p27 (Figure 4a), as expected [22,23,24,25], even at low concentration of miR-155 (Figure S5a). On the other hand, overexpression of miPEP155 did not induce a decrease in the expression of miR-155 targets (Figure 4b), confirming the results obtained above. In fact, we observed an increased expression of Rictor and EGFR upon overexpression of WT miPEP155 when compared to mutant miPEP155 (Figure 4b). Similarly, while overexpression of miR-497 led to downregulation of genes encoding cell cycle activators, as expected [26,27], such as CDC25A, CDK6, and Cyclin E (Figure 4c), overexpression of miPEP497 had no effect on CDC25A and Cyclin E levels, although a slight decrease in CDK6 levels was observed (Figure 4d). Thus, these results indicate that miPEP155 and miPEP497 do not regulate the levels of their own pri-miRNAs and consequently the activity of the processed miRNAs. 

### 2.3. Overexpression of miPEP200a Does Not Affect the Activity of miR-200a or miR200b

**Figure 5:**
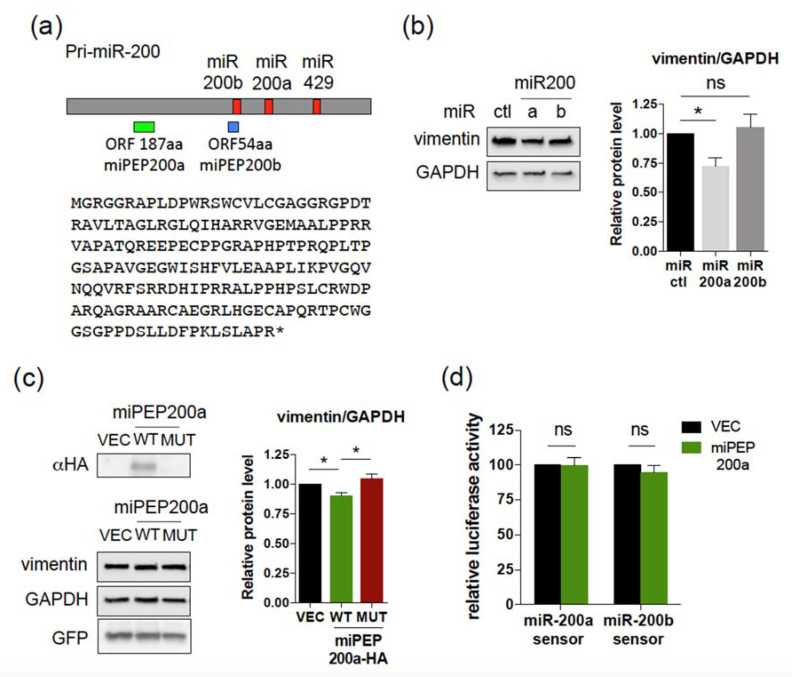
miPEP200a does not regulate the activities of miR-200a and miR-200b. (**a**) Schematic structure of human pri-miR-200 with the miRNAs (red boxes), the 54 amino acid sORF (miPEP200b, blue box), the 187 amino acid sORF (green box), and miPEP200a sequence indicated. (**b**,**c**) Western blot analysis of PC3 cells transfected with control miRNA or miR-200a or miR-200b (10 nM) **(b)** or control vector or vectors expressing HA-tagged WT or mutated miPEP200a. (**c**) The vectors contain the EGFP gene, used as control of transfection efficiency. GAPDH was used as loading control. Quantifications of the immunoblots are presented in the right panels. Data are means ± S.E.M. of four (**b**) and seven (**c**) independent experiments. (**d**) Endogenous miR-200a and miR-200b activities upon overexpression of miPEP200a into PC3 cells determined by dual luciferase assays 48 h post-transfection. The relative luciferase activities of miPEP200a transfected cells were compared to that of vector transfected cells, set to 100. Data are means ± S.E.M. of four independent experiments. * *p* < 0.05.

Recently, two sORFs have been identified within the human pri-miRNA of miR-200a and miR-200b, a 187 amino acid ORF (coding miPEP200a) and a 54 amino acid ORF (coding miPEP200b), and overexpression of these HA-tagged-miPEPs in PC3 prostate cancer cells inhibited their migration and downregulated the vimentin expression [12]. Since miR-200a and miR-200b also regulate cell migration [13], it was suggested that the miPEPs200 might function by activating miR-200a and miR-200b. To investigate this point, and according to the schematic structure described in Fang et al., (2017), we identified a 187 amino acid peptide encoded by a sORF of 564 nt located in the 5′ part of pri-miR-200 (Figure 5a), likely corresponding to miPEP200a. Overexpression of miR-200a or miR-200b in PC3 cells revealed that only miR-200a was able to downregulate vimentin expression (Figure 5b). Overexpression of miPEP200a in PC3 cells slightly decreases vimentin expression (Figure 5c). However, co-transfection of the miR-200a or miR-200b luciferase reporters together with a control vector or the miPEP200a expression construct showed that miPEP200a overexpression did not change endogenous miR-200a or miR-200b activities (Figure 5d). On the other hand, co-transfection of the miR-200a or miR-200b luciferase reporters together with a control miRNA or their corresponding miRNAs showed a strong reduction in luciferase activities with the miRNAs relative to control miRNA, validating our miRNA sensors (Figure S3c,d). These results suggest that miPEP200a downregulates vimentin expression independently of the miR200a and miR200b activation. 

## 3. Discussion

Some miRNAs are located in RNA precursors (pri-miRNAs) annotated as lncRNAs, as they lack long ORFs and were thought to be unable to encode proteins. However, recent studies have shown that a few lnc pri-miRNAs encode small peptides called miPEPs (miRNA-encoded peptides). Initially discovered in plants [7], miPEPs encoded by the lncRNAs MIR22HG (pri-miRNA-22) [11], MIR155HG (pri-miR-155) [15], and MIR34AHG (pri-miR34a) [16] have also been identified in humans. Analysis of the 48 lnc MIRHGs identified in UCSC Genome Browser with the GWIPS-viz genome browser [17] revealed that 32 of them contain sORFs exhibiting ribosome profiling marks (Table S1). Moreover, using OpenProt (an online database containing predicted ORFs longer than 30 codons supported by ribosome profiling and mass spectrometry studies from several species, tissues, and cell lines (www.openprot.org, (accessed on 4 March 2021)) (release 1.5, 23 June 2020) [28]) revealed that 22 lnc MIRHGs encode peptides identified by mass spectrometry (Table S2), expanding the number of potential miPEPs in humans.

So far, the only known function of plant miPEPs is to enhance the transcription of their own pri-miRNAs, thereby increasing the level and activity of their associated miRNAs [7,8]. Concerning human miPEPs, except for the peptide C17ORF91 encoded by MIR22HG lncRNA whose function has not been investigated [11], a role for miPEP155, encoded by MIR155HG [15], and miPEP133, encoded by MIR34AHG [16], has been established. MiPEP133 is mainly localized in the mitochondria, where it interacts with mitochondrial heat shock protein 70 (mtHsp70 or HSPA9) and decreases binding of mtHSP70 to its partners. In this way, miPEP133 disrupts mitochondrial function, which activates p53 transcriptional activity. Since expression of MIR34AHG is regulated by p53 [29,30,31], miPEP133-induced p53 activation leads to increased expression of pri-miR-34a/miR-34a, likely amongst a plethora of other targets. Thus, although miPEP133 is involved in a positive feedback regulation of its pri-miRNA, the mechanism appears to be different from that observed with plants miPEPs. Indeed, for these, the nuclear localization suggests a more direct implication, and there were shown to be specific activators of their own miRNA genes, while activation of p53 by miPEP133 probably leads to induction of many other genes, including miRNAs, since p53 regulates the expression of various miRNAs [31].

It remains unclear whether the regulation of miRNA gene expression by plant miPEPs and human miPEP133 is a general rule of human miPEPs such as miPEP155 or others. MiPEP155 was shown to regulate antigen presentation in dentritic cells [15]. Indeed, miPEP155 binds to HSC70, a chaperone required for antigen trafficking and presentation, and impairs antigen transport in these cells by disrupting the HSC70-HSP90 complex. MiPEP155 is also expressed in non-dendritic cells, since it was also detected in HEK293T cells [15], and our study shows that the spliced pri-miR-155 transcript is able to express miPEP155 in Hela cells, which is in agreement with the ribosome profiling marks detected in these cells [20]. However, our experiments show that overexpression of miPEP155 in Hela cells has no impact on the expression of pri-miR155 and on the activity of miR-155. To consolidate our results, we analysed RNA-Seq experiments performed in THP-1-derived dendritic cells treated with miPEP155 or a scramble peptide [15]. In these experiments, MIR155HG expression is not affected after miPEP155 treatment (0.065 log2 fold-change between miPEP155 versus scramble peptide treatment). Our results are consistent with the idea that miPEP155 has an antagonistic role to miR-155. Indeed, miPEP155 exhibits an anti-inflammatory role [15], while miR-155 mediates inflammation and immune response [14,32]. Moreover, we found that miPEP155 overexpression in Hela cells increases Rictor and EGFR expression, while overexpression of miR-155 represses these genes.

To extend our observation, we tested others human miPEPs. We chose MIR497HG, which exhibits a simple structure and is expressed as unspliced and spliced transcripts. The unspliced transcript corresponds to pri-miR-497, and we found that this pri-miRNA contains a functional ORF of 66 nt located upstream of the miR-497 that can be expressed as a peptide of 21 amino acids (miPEP497) in Hela cells, in agreement with the ribosome profiling marks detected at this ORF. Interestingly, both miPEP155 and miPEP497 are well conserved in primates and mice at the amino acid levels, suggesting a conserved function in various species. In this regard, miPEP155 can also interact with mouse HSC70 and possesses an anti-inflammatory role in this species [15]. Like miPEP155, overexpression of miPEP497 does not affect the levels of pri-miR497 and the activity of miR-497. Thus, from our results we conclude that miPEP155 and miPEP497 do not regulate the levels of their own pri-miRNAs and consequently the activity of the processed miRNAs. We also found that miPEP200a, a sORF-encoded peptide identified within human pri-miRNA of miR-200a and miR-200b, does not impact the activity of miR-200a and miR-200b, suggesting that the downregulation of vimentin expression observed after overexpression of this miPEP is independent of the miR-200 pathway.

Altogether, these results lead us to conclude that the positive feedback regulation of miPEPs toward their pri-miRNAs observed in various plant species and with human miPEP133 is not a general rule of miPEPs function. Further studies are needed to test whether miPEPs other than miPEP133 could regulate their pri-miRNAs/miRNAs

## 4. Materials and Methods

### 4.1. Cell Culture and Transfections

Hela cells from ATCC and PC3 cells were grown at 37 °C with 5% CO_2_ in DMEM (Hela) or RPMI-1640 (PC3) (Gibco, LifeTechnologies, Carlsbad, CA, USA) supplemented with 10% fetal bovine serum and 2 µg/mL penicillin/streptomycin (Sigma-Aldrich, St Louis, MO, USA). For transfection of miRNAs, cells were seeded at a density of 1.2 × 10^5^ (Hela) or 1.7 × 10^5^ (PC3) into 6-well plates the day before transfection to reach 30–50% confluence the day of transfection. For transfection of plasmid vectors, cells were seeded at a density of 3 × 10^5^ (Hela) or 3.5 × 10^5^ (PC3) into 6-well plates the day before transfection to reach 70% confluence the day of transfection. Transfection of miRNAs or plasmid vectors were performed respectively with Interferin or JetPrime reagents (Polyplus transfection, Illkirch, France) following the manufacturer’s instructions. Cells were harvested 48 h post-transfection, and RNA and protein extractions were performed. 

### 4.2. Immunoblotting and Antibodies

Cells were lysed in NuPAGE® LDS Sample Buffer (Novex, Life Technologies, Carlsbad, CA, USA), sonicated, and boiled at 96 °C for 3 min. Proteins were separated on 4–15% SDS-PAGE gels (BioRad, Hercules, CA, USA) and transferred to polyvinylidene difluoride membrane (Immobilon-P, Millipore, Burlington, MA, USA). Membranes were blocked in TBS-T (Tris Buffered Saline with 0.1% Tween 20) containing 5% non-fat dry milk, blotted with antibodies overnight at 4 °C, washed, then incubated at room temperature with HRP-conjugated secondary antibodies (Jackson ImmunoResearch, Ely, UK). After washes, detection was achieved with chemiluminescence detection reagents (Clarity^TM^ Western ECL substrate from Bio-Rad). Image acquisitions and quantifications of immunoblots were performed with a Fusion Solo X chemiluminescence imaging system using the Evolution-Capture software (Vilber Lourmat, Marne La Vallée, France) or with a ChemiDoc Touch imaging system using the Image Lab Touch software (BioRad). Monoclonal antibodies against Rictor (H11), C/EBPß (H-7), K-Ras (F234), p27 (sx53G8.5), GFP (B-2), CDC25A (F6), CyclinE (HE12), ß-actin (C4), and polyclonal antibodies against CDK6 (C21) were purchased from Santa Cruz Biotechnology (Santa Cruz, CA, USA). Rabbit anti-EGFR and anti-HA (C29F4) were from Cell Signaling Technology (Beverly, MA, USA), rabbit anti-turbo GFP (AB514) from Evrogen (Moscow, Russia), mouse anti-vimentin from BD Pharmingen (San Diego, CA, USA), mouse anti-vinculin and anti-ß-tubulin from Sigma, mouse anti-GAPDH (6C5) from Thermo Fisher Scientific (Waltham, MA, USA), and anti-miPEP155 (affinity-purified polyclonal rabbit antibodies raised against the 17 amino acid sequence of the miPEP155) from Genscript (Piscataway, NJ, USA). 

### 4.3. Plasmids and miRNAs

The pcDNA3 EGFP and pcDNA3 turboGFP were constructed by substituting the neomycin gene of pcDNA3 (Invitrogen, Carlsbad, CA, USA) with the EGFP or turboGFP genes, respectively. The pri-miR-155 (FL-BIC), obtained from A. van den Berg and described in [18], and pri-miR-497 (isolated from BJ-hTert) were cloned in the pcDNA3 EGFP. The pri-miR-155 and pri-miR-497 containing the sORFs fused in frame with EGFP were cloned in the pcDNA3 turboGFP. The miPEPs expression constructs were obtained by cloning the sORFs placed downstream from a Kozak consensus sequence in the pIRES2 turboGFP (constructed by replacing the EGFP gene of pIRES2 EGFP vector—from Clontech—with the turboGFP gene) (for miPEP155) or pcDNA3 EGFP (for miPEP497 and miPEP200a). The miR sensor plasmids were constructed by cloning three repeat reverse complementary sequences corresponding to miR-155-5p or miR-497-5p or miR-200a-3p or miR-200b-3p (synthesized by Eurofins Genomics, Ebersberg, Germany) into the end of Renilla luciferase of the psiCHECK-2 dual luciferase reporter vector (Promega, Madison, WI, USA). The hsa-miR-155-5p, hsa-miR-497-5p, hsa-miR-200a-3p, and hsa-miR-200b-3p pre-miR miRNA precursors, pre-miR negative control #2, hsa-miR-155-5p, hsa-miR-497-5p miRVana miRNA inhibitors, and anti-miR negative control #1 were purchased from Ambion (Thermo Fisher Scientific). 

### 4.4. Reverse Transcription (RT) and Quantitative Polymerase Chain Reaction (qPCR)

Total RNA of cultured cells was extracted using Tri-Reagent (Sigma) and digested with DNase (Thermo Fisher Scientific). Total RNA (800 ng) was reverse-transcribed for cDNA synthesis using Maxima Reverse Transcriptase kit (Thermo Fisher Scientific) following the manufacturer’s instructions, and qPCR was performed using the SsoAdvanced Universal SYBR Green Supermix (BioRad) on a CF×96 real time system device (BioRad) according to the manufacturer’s instructions. All samples were analyzed using the CFX Manager Software (BioRad). The mRNA levels were normalized to GAPDH. Primers for qPCR were the following: 

pri-miR-155 Forward 5′-GGGAGGATGACAAAGAAGCA-3′, Reverse 5′-TGAACATCC

CAGTGACCAGA-3′; 

miPEP155 Forward 5′-TGGAGATGGCTCTAATGGTG-3′, Reverse 5′-AACCACAGATT TCCCCTTCC-3′; 

pri-miR-497 Forward 5′-TCTGACTGGGAGTGGAGGAAC-3′, Reverse 5′-CACATTTGG GGTGCAGGAGAA-3′; 

miPEP497 Forward 5′-ATGGGCTGGGACGGGTTT-3′, Reverse 5′-AGGGGTTCCTC CACTCCC-3′; 

GAPDH Forward 5′-GAAGGTGAAGGTCGGAGTCA-3′, Reverse 5′-GAAGATGGTGAT

GGGATTTC-3′. Results were analysed using the 2-∆∆Ct method.

### 4.5. Dual Luciferase Reporter Assays 

Hela or PC3 cells were seeded in 12-well plates to reach 70% confluence the day of transfection, and then transfected with the luciferase sensors of miRNA activity (psiCHECK2) (25 ng), with either the miRNA control or the specific pre-miR miRNA precursors (0.1, 1 or 10 nM), or the empty vector or miPEP expressing vectors (250 ng). Transfected cells were lysed 48h after transfection, and luciferase activities were assayed by a Dual-Luciferase Reporter Assay System (Promega) according to the manufacturer’s instructions, with a Luminoskan and Skanlt^Tm^ software for microplate readers (Thermo Scientific). The data were analyzed by normalizing Renilla luciferase activity (which quantifies the miRNA activity) with Firely luciferase activity (to monitor the transfection efficiency) and the ratio of Renilla luciferase activity to Firefly luciferase activity was calculated to indicate the activity of the reporter. 

### 4.6. Statistical Analyses

Data are shown as means ± SEM and were considered statistically significant at *p* < 0.05. GraphPad Prism software (version 6, GraphPad Software Inc., San Diego, CA, USA) was used for analysis. For statistical analysis, Mann–Whitney or one sample *t*-test was used. * *p* < 0.05, ** *p* < 0.01, and *** *p* < 0.001, ns: non-significant. 

## Data Availability

The data presented in this study are openly available in http://gwips.ucc.ie (accessed on 4 March 2021) at doi:10.1093/nar/gkt1035, reference number [17]; in www.openprot.org (accessed on 4 March 2021) at doi:10.1093/nar/gky936, reference number [28].

## Supplementary Materials

The following are available online at https://www.mdpi.com/article/10.3390/ijms22073432/s1, Figure S1: Identification of ribosome profiling marks within M155HG and MIR497HG, Figure S2: Alignment of the miPEP155 and miPEP497 peptidic sequences in primates and mouse, Figure S3: Validation and sensitivity of the luciferase sensors of miR activity, Figure S4: Validation of anti-miRNAs and Hela cells to study miR-155 and miR-497, Figure S5: Expression of miR-155 and miR-497 target genes upon overexpression of miR-155 and miR-497, Table S1: List of lnc MIRNA host genes and their encoded-miRNAs containing sORFs exhibiting ribosome profiling marks, Table S2: List of lnc MIRNA host genes and their encoded-miRNAs containing sORFs-encoded peptides/proteins with experimental evidence obtained from mass spectrometry.
