# Supplementary material for: Evidence That Regulation of Pri-miRNA/miRNA Expression Is Not a General Rule of miPEPs Function in Humans
Source: Int J Mol Sci. 2021 Mar 26;22(7):3432. doi: 10.3390/ijms22073432 (PMC8038077; doi:10.3390/ijms22073432)
Supplement: Supplementary file 1 [file ijms-22-03432-s001.pdf]

Supplementary Figure 1

a

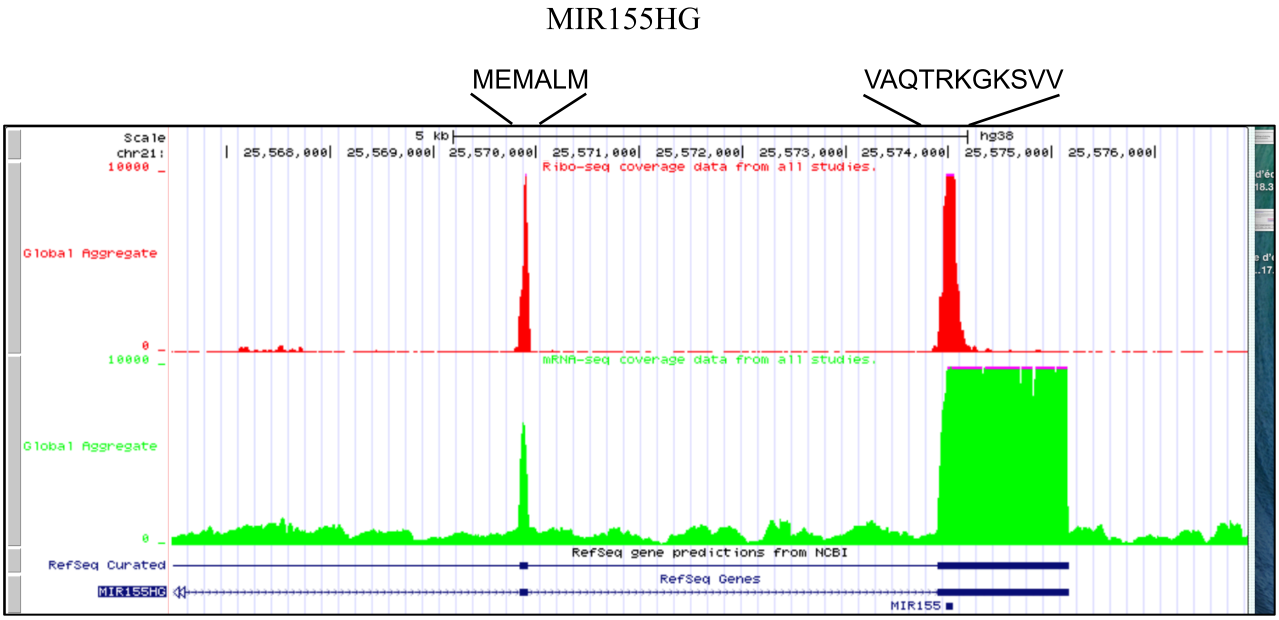

b

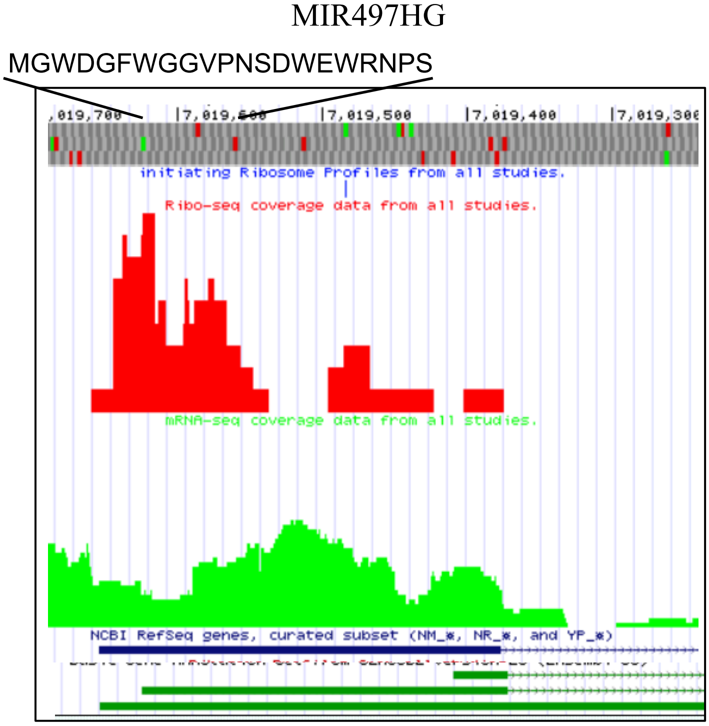

Figure S1: Identification of ribosome profiling marks within M155HG and MIR497HG

Ribosome profiling (red) and mRNA-seq coverage (green) for MIR155HG **(a)** and the 5' part of MIR497HG **(b)** with the sequences of the miPEPs indicated. Analyse was done using the GWIPS-viz browser (http://gwips.ucc.ie) [15]

Supplementary Figure 2

miPEP155

Human	MEMALMVAQTRKGKSVV*
Chimpanzee	MEMALMVAQTRKGKSVV*
Gorilla	MEMALMVAQTRKGKSVV*
Orangutan	MEMAVMVAQTRKGKSVV*
Macaque	MEMALMVAQTRKGKSVV*
Baboon	MEMALMVAQTRKGKSVV*
Marmoset	KEMALRVAQTRKGKSVV*
Tarsier	KEMLLMVAQTNKEKSVV*
Mouse	EELVLMVLQTRKGKCVV*

miPEP497

Human	MGWDGFWGGVPNSDWEWRNPS*
Chimpanzee	MGWDGFWGGVPNSDWEWRNPS*
Gorilla	MGWDGFWGGVPNSDWEWRNPS*
Orangutan	MGWDGFWGGVPNSDWEWRNPS*
Macaque	MGWDGFWGGVPNSDWEWRNPS*
Baboon	MGWDGFWGGVPNSDWEWRNPS*
Marmoset	MGWDGFWGGVPNSDWEWRNPS*
Tarsier	MGWDGFWGGVPNSDWEWRNPS*
Mouse	MGWDGFWGGVPNSDWEWRNPSR…

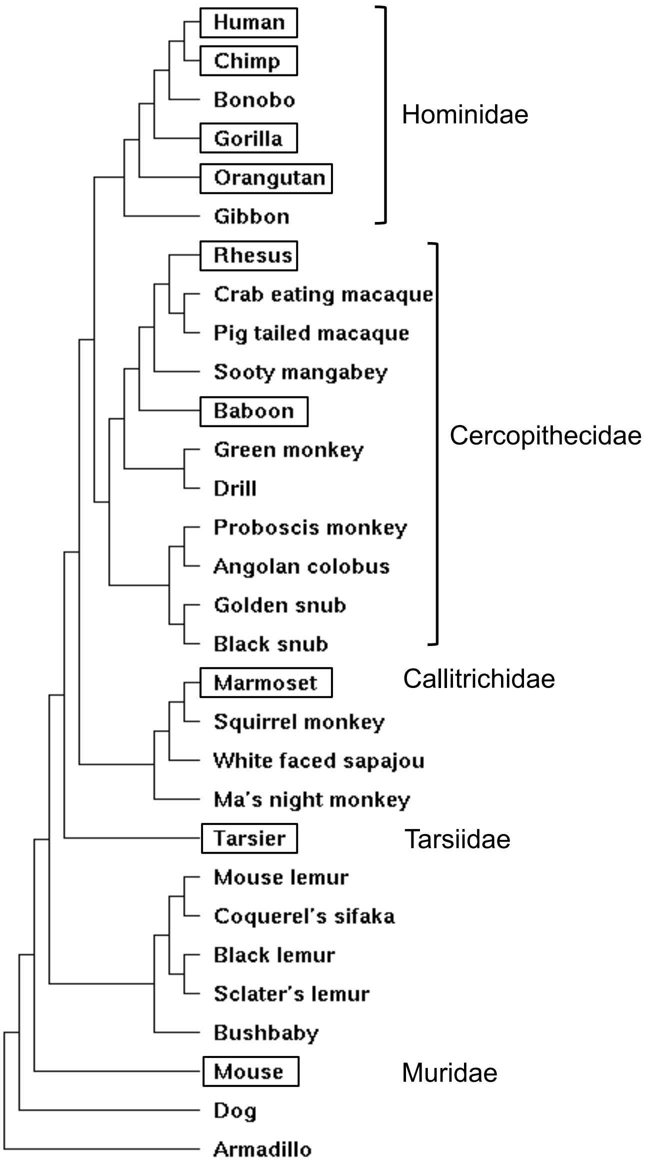

Figure S2: Alignment of the miPEP155 and miPEP497 peptidic sequences in primates and mouse.
The right panel shows the phylogenetic tree of the species analyzed

Supplementary Figure 3

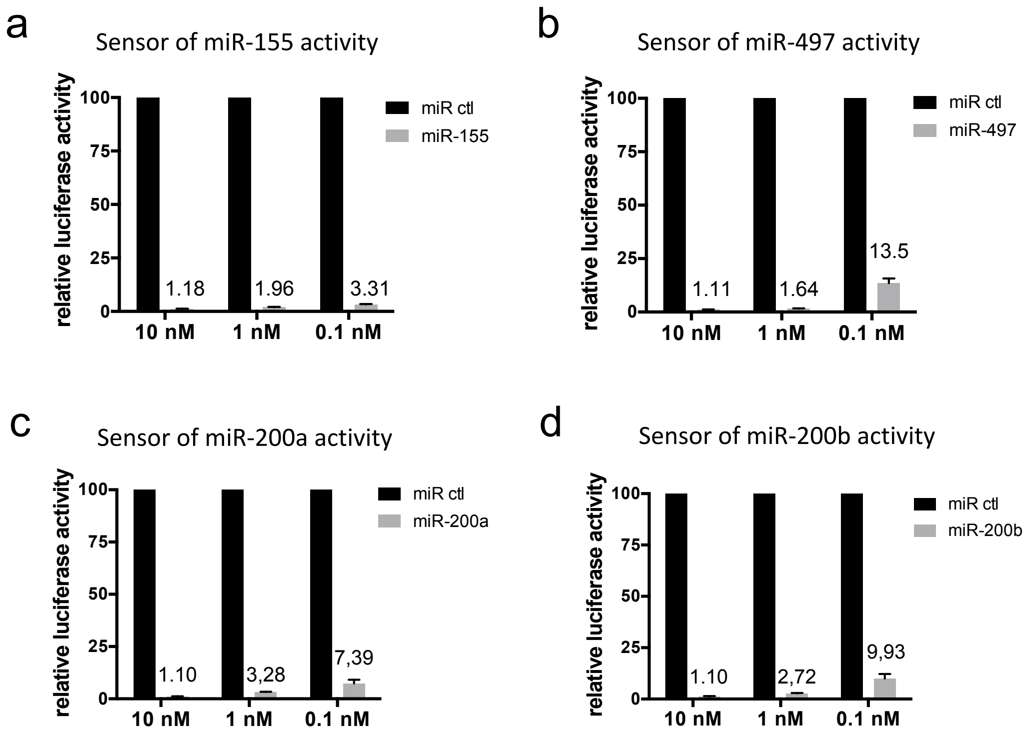

Figure S3: Validation and sensitivity of the luciferase sensors of miR activity.
Hela **(a, b)** or PC3 **(c, d)** cells were cotransfected with the luciferase sensors of miR activity together with decreasing amounts of miR control (ctl) or miR. Transfected cells were harvested 48 h post-transfection for dual luciferase assays. The relative luciferase activities of miR transfected cells were compared to that of the miR ctl transfected cells, set to 100. Graphs show means of three independent experiments.

Supplementary Figure 4

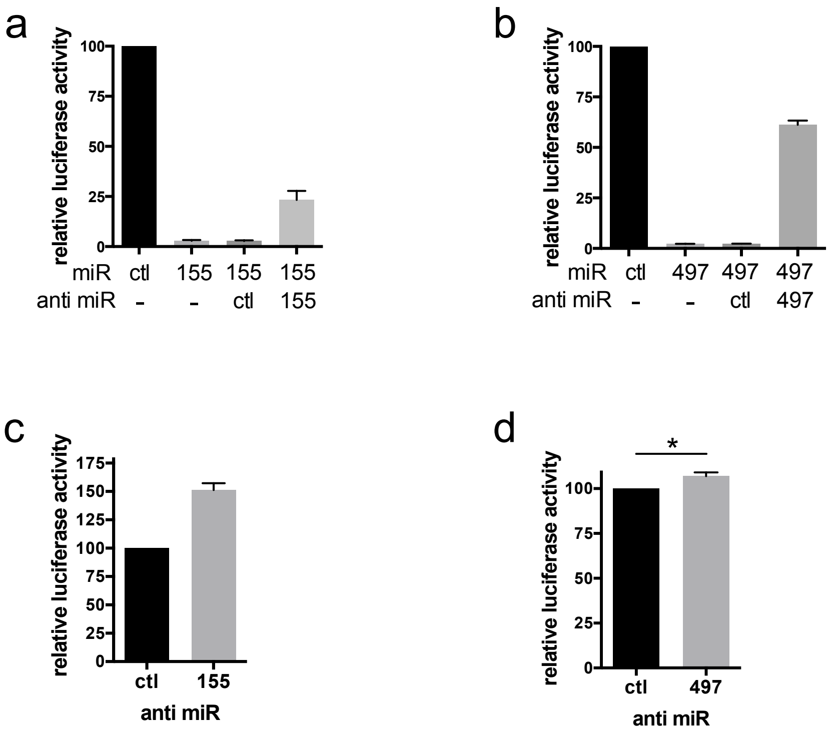

Figure S4: Validation of anti-miRNAs and Hela cells to study miR-155 and miR-497
(a, b) Anti miRNAs rescue miRNAs overexpression. Hela cells were cotransfected with miR control (ctl), miR-155 **(a)** or miR-497 (1 nM) **(b)**, with or without anti miR ctl, anti miR-155 **(a)** or anti miR-497 (10 nM) **(b)**.
(c, d) Hela cells were transfected with anti miR ctl (10 nM) **(c)**, anti miR-155 (10 nM) or anti miR-497 (10 nM) **(d)**. Transfected cells were harvested 48 h post-transfection for dual luciferase assays**.** The relative luciferase activities of miR or anti-miR transfected cells were compared to that of the miR ctl or anti miR ctl transfected cells, set to 100. Graphs show means ± SEM of three **(a, b, c)** or five **(d)** independent experiments; * $p<0.05$.

Supplementary Figure 5

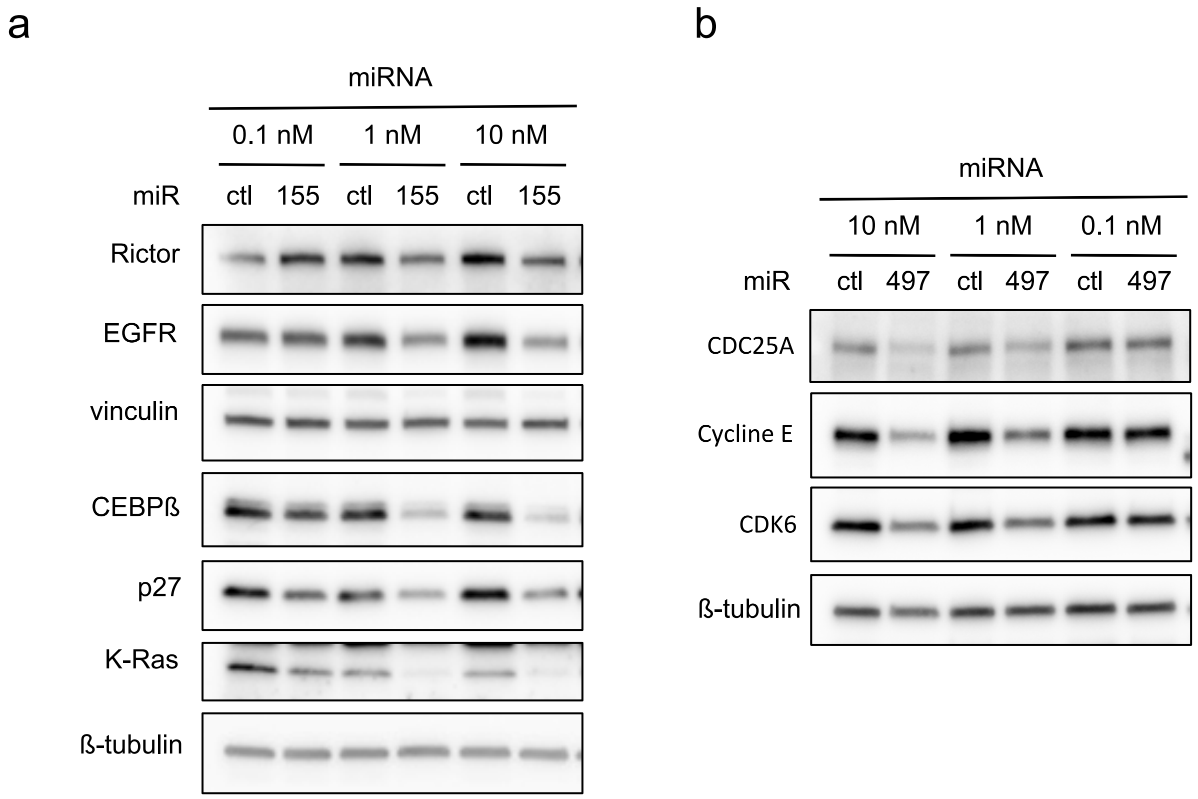

Figure S5: Expression of miR-155 and miR-497 target genes upon overexpression of miR-155 and miR-497

Hela cells were transfected with increasing amounts of miR control (ctl), miR-155 **(a)** or miR-497 **(b)** and 48 h later subjected to immunoblot analyses using the indicated antibodies. Vinculin and ß-tubulin were used as loading control.

Table S1

List of lnc MIRNA Host Genes and their encoded-miRNAs containing sORFs exhibiting ribosome profiling marks after analyses with the GWIPS-viz genome browser (http://gwips.ucc.ie) [15]. The MIR Host genes for which a miPEP was co-detected by mass spectrometry (Table S2) are listed in bold.

encode id	MIR Host gene	MIR hosted
**ENSG00000255248**	**MIR100HG**	**MIR-100-LET-7a-2 cluster MIR125B-1**
**ENSG00000254377**	**MIR124-2HG**	**MIR124-2**
ENSG00000225206	MIR137HG	MIR137 MIR2682
ENSG00000234883	MIR155HG	MIR155
ENSG00000215417	MIR17HG	MIR17-92 cluster
**ENSG00000229989**	**MIR181A1HG**	**MIR181A1 MIR181B1**
ENSG00000224020	MIR181A2HG	MIR181A2 MIR181B2
**ENSG00000262454**	**MIR193BHG**	**MIR193B MIR365A**
**ENSG00000229719**	**MIR194-2HG**	**MIR194-2 MIR192**
ENSG00000186594	MIR22HG	MIR22
ENSG00000282810	MIR210HG	MIR210
**ENSG00000254349**	**MIR2052HG**	**MIR2052**
**ENSG00000253522**	**MIR3142HG**	**MIR3142 MIR146A**
ENSG00000171889	MIR31HG	MIR31
**ENSG00000280870**	**MIR325HG**	**MIR325 MIR384**
**ENSG00000228526**	**MIR34AHG**	**MIR34A**
ENSG00000224184	MIR3681HG	MIR3681
ENSG00000251230	MIR3945HG	MIR3945
**ENSG00000172965**	**MIR4435-2HG**	**MIR4435-2**
ENSG00000247516	MIR4458HG	MIR4458
ENSG00000228824	MIR4500HG	MIR4500
**ENSG00000267532**	**MIR497HG**	**MIR497 MIR195**
ENSG00000223749	MIR503HG	MIR503
ENSG00000224141	MIR548XHG	MIR548X
ENSG00000229401	MIR5689HG	MIR5689
**ENSG00000228340**	**MIR646HG**	**MIR646**
ENSG00000176840	MIR7-3HG	MIR7-3
ENSG00000260083	MIR762HG	MIR762
ENSG00000255571	MIR9-3HG	MIR9-3
ENSG00000267374	MIR924HG	MIR5583-1 MIR5583-2 MIR924
**ENSG00000215386**	**MIR99AHG**	**MIR99A MIRLET7C MIR125B2**
**ENSG00000197182**	**MIRLET7BHG**	**MIR3619 MIRLET7A3 MIR4763 MIRLET7B**

Table S2
List of lnc MIRNA Host Genes and their encoded-miRNAs containing sORFs-encoded peptides/proteins with experimental evidence obtained from mass spectrometry (www.openprot.org) [26]. The MIR Host genes for which a miPEP was co-detected in Riboprofiling experiments (Table S1). are listed in bold

encode id	MIR Host gene	MIR hosted
**ENSG00000255248**	**MIR100HG**	**MIR-100-LET-7a-2 cluster MIR125B-1**
**ENSG00000254377**	**MIR124-2HG**	**MIR124-2**
ENSG00000265142	MIR133A1HG	MIR1-2 MIR133A1
**ENSG00000229989**	**MIR181A1HG**	**MIR181A1 MIR181B1**
**ENSG00000262454**	**MIR193BHG**	**MIR193B MIR365A**
**ENSG00000229719**	**MIR194-2HG**	**MIR194-2 MIR192**
**ENSG00000254349**	**MIR2052HG**	**MIR2052**
ENSG00000226702	MIR217HG	MIR216B, 216A, 217
**ENSG00000253522**	**MIR3142HG**	**MIR3142 MIR146A**
**ENSG00000280870**	**MIR325HG**	**MIR325 MIR384**
**ENSG00000228526**	**MIR34AHG**	**MIR34A**
ENSG00000245832	MIR4300HG	MIR4300
**ENSG00000172965**	**MIR4435-2HG**	**MIR4435-2**
ENSG00000280237	MIR4697HG	MIR4697
**ENSG00000267532**	**MIR497HG**	**MIR497 MIR195**
ENSG00000236901	MIR600HG	MIR600
**ENSG00000228340**	**MIR646HG**	**MIR646**
ENSG00000227195	MIR663AHG	MIR663A
ENSG00000236172	MIR7515HG	MIR1515
**ENSG00000215386**	**MIR99AHG**	**MIR99A MIRLET7C MIR125B2**
ENSG00000197182	MIRLET7BHG	MIR3619 MIRLET7A3 MIR4763 MIRLET7B
**ENSG00000230262**	**MIRLET7DHG**	**MIRLET7D**
